# Retraction of ‘Protein kinase CK2 phosphorylation regulates the interaction of Kaposi's sarcoma-associated herpesvirus regulatory protein ORF57 with its multifunctional partner hnRNP K’

**DOI:** 10.1093/nar/gkac574

**Published:** 2022-07-04

*Nucleic Acids Research*, Volume 32, Issue 18, 15 September 2004, Pages 5553–5569, https://doi.org/10.1093/nar/gkh876

Following allegations of image manipulation in Figures 1A, 6C, 8B, 8C, and 8E in 2021, the journal conducted a brief investigation, referred the matter to the authors’ institution, and published an Expression of Concern. In May 2022, the institutional panel investigating the allegations concluded the figures are not authentic and the scientific integrity of the article is compromised, and they recommended retracting the article. Their report includes: ‘On the balance of probabilities, the Panel believe that these data as presented in the publication have been inappropriately manipulated. As such, the data and its interpretation are misleading and unreliable.’ The Editors of the journal are, therefore, now retracting this article.

